# Items

**Published:** 1874-08

**Authors:** 


### ITEMS.

After the first of September a change is to be made in the Medical Board of Bellevue Hospital. By resolution of the Commissioners of Public Charities and Corrections, the Board from that date is to consist of Profs. Austin Flint, Sr. and James R. Wood, of Bellevue College ; Alonzo Clark and Henry B. Sands, of the College of Physicians and Surgeons ; Alfred L. Looms and Stephen Smith, of the Medical Department of the N. Y. University, and Drs. Wm. B. Eager and Ernst Krackowizer, who are connected with no school. These are to nominate eleven others to be confirmed by the Commissioners, to constitute, with themselves, the Medical Board of Bellevue Hospital. This will give each school in New York an equal number of representatives in the Staff of the Hospital.——Messrs. G. P. Putnam's Sons announce that, on the first of October next, they will issue the first number of a quarterly Journal to be called The Archives of Dermatology. The Archives will be under the Editorial supervision of Dr. L. D. Buckley of New York, who has made several valuable contribution to Dermatology, and has already attained a high position in his specialty. We wish for him and the publishers the complete success of their undertaking.——Prof. Frank H. Hamilton offers a prize of one hundred dollars for seven cases of fracture of the shaft of the femur, occurring in persons over twenty yaars of age, who were not at the time of the injury suffering from paralysis or atrophy of the limbs, which have been treated by the surgeon presenting the cases, and which have united without shortening. The diagnosis of the fracture to be verified by at least one other surgeon who was present at or near the time of the accident, and who assisted in making the diagnosis. The measurement to be made by Dr. Hamilton and two other surgeons, to be chosen by himself and the person presenting the cases.——Owing to ill health, Prof. Hughes Bennett has resigned the chair of Institutes of Medicine in the University of Edinburgh.——We present the following as a sample of scientific (?) Homœopathy, taken from the proceedings of the Loraine Co., Ohio, Medical Society : "Dr. Park reported in writing a case of hepatization of the left lung, in a girl six years of age, cured with Lycopodium. Dr. Wolcott thought lycop. acted best in troubles of the right lung. Drs. Starkey and Hayward thought the left lung more easily affected by this remedy." And still these men make a claim of being scientific physicians.——Our July number was detained by a mistake in binding.

